# The Surgical Timing Gap in Elderly Hip Fracture Management: A Regional Systems Analysis

**DOI:** 10.7759/cureus.115388

**Published:** 2026-08-29

**Authors:** Abdelrahim Shaaeldin, Osama F Almaghthawi, Leena M. Salem, Alhassan Yousef Alturkestani, Raghad A Sokail, Nesreen M Abujabal, Eilaf H Hawsah, Fatima J Alnakli, Dina Algheryafi, Lena F Alahmadi

**Affiliations:** 1 Orthopedics and Traumatology, Al-Rayan College of Medicine, Medina, SAU; 2 Medicine, Ministry of Health, Medina, SAU; 3 Orthopedic Surgery, Faculty of Medicine, Al-Rayan College of Medicine, Medina, SAU; 4 Emergency Medicine, College of Medicine, Ministry of Health, Medina, SAU; 5 College of Medicine, Al-Rayan College of Medicine, Medina, SAU; 6 Orthopedics, Al-Rayan College of Medicine, Medina, SAU; 7 Orthopedics, King Fahad General Hospital, Medina, SAU; 8 Orthopedic Surgery, Al-Rayan College of Medicine, Medina, SAU; 9 Medicine and Surgery, Al-Rayan College of Medicine, Medina, SAU; 10 Medicine, Al-Rayan College of Medicine, Medina, SAU

**Keywords:** elderly, health systems, hip fracture, length of stay, saudi arabia, surgical delay

## Abstract

Background: Timely surgical management of hip fractures in elderly patients is a key determinant of outcomes. International guidelines recommend early surgery; however, data on system-level contributors to delay in Saudi Arabia remain limited.

Study aim: The aim of this study is to evaluate surgical timing in elderly hip fracture patients in the Medina region, identify patient-, provider-, and system-level factors contributing to delay, and assess the impact of delayed surgery on clinical outcomes.

Methods: This retrospective cohort systems analysis included 400 patients aged ≥60 years admitted with femoral neck, intertrochanteric, or subtrochanteric hip fractures to two regional hospitals. Patients were stratified into early (≤48 hours) and delayed (>48 hours) surgery groups. Descriptive statistics, bivariate analyses, and multivariable logistic regression were performed to identify predictors of delay. Outcomes included length of stay, postoperative complications, and in-hospital mortality.

Results: Only 22.0% of patients underwent surgery within 48 hours. The median time to surgery was 88.2 hours. Delayed surgery was associated with older age (p = 0.052) and higher ASA class (p = 0.029). Weekend admission (31.7% vs. 14.0%; p = 0.009), a greater number of preoperative consultations (p = 0.002), and operating room list deferral (25.2% vs. 0%; p < 0.001) were significantly associated with delay. In multivariable analysis, independent predictors of delay included older age (aOR 1.04; p = 0.015), intertrochanteric fracture (aOR 2.07; p = 0.048), weekend admission (aOR 2.84; p = 0.029), longer laboratory turnaround time (aOR 1.02; p = 0.012), and increased number of consultations (aOR 1.88; p < 0.001). Delayed surgery was associated with a longer hospital length of stay (median 8 vs. 6 days; p < 0.001), while postoperative complications (p = 0.312) and in-hospital mortality (p = 0.527) did not differ significantly between groups.

Conclusion: Surgical delay in elderly hip fracture patients in the Medina region is common and is driven predominantly by modifiable system-level factors rather than patient-related clinical instability. Addressing organizational barriers, particularly weekend service gaps, consultation burden, and operating room access, may substantially improve timeliness and efficiency of hip fracture care.

## Introduction

Hip fractures represent a major public health challenge worldwide and are among the most serious injuries affecting the elderly population [1-3]. With increasing life expectancy and population aging, the global incidence of hip fractures is projected to rise substantially over the coming decades, placing significant pressure on healthcare systems [4-6]. Elderly patients sustaining hip fractures experience high rates of morbidity, functional decline, institutionalization, and mortality, with reported one-year mortality rates ranging between 20% and 30% despite advances in surgical and perioperative care.

Timely surgical management is a cornerstone of evidence-based hip fracture care. Delays to surgery have been consistently associated with adverse outcomes, including prolonged immobilization, increased risk of medical complications such as pneumonia and thromboembolism, longer hospital length of stay, and higher short- and long-term mortality [7-9]. As a result, international clinical guidelines emphasize early operative intervention as a key quality indicator. The National Institute for Health and Care Excellence (NICE) recommends surgery on the day of, or the day following, admission whenever medically feasible, while British Orthopaedic Association Standards for Trauma (BOAST) and the Association of Anaesthetists advocate surgery within 36 hours for most patients [10,11].

Despite strong evidence and clear guideline recommendations, delays to hip fracture surgery remain common in routine clinical practice. Importantly, accumulating literature suggests that many of these delays are not attributable to unavoidable medical instability but rather to modifiable system-level and organizational factors, including operating room availability, weekend staffing patterns, diagnostic and laboratory delays, and fragmented preoperative decision-making processes [12,13]. Health systems that have implemented structured hip fracture pathways, characterized by multidisciplinary coordination, orthogeriatric co-management, protected trauma operating lists, and standardized protocols, have demonstrated significant improvements in time to surgery, length of stay, and patient outcomes [14-16].

In the Middle East and Saudi Arabia, hip fractures constitute a growing clinical burden due to demographic shifts and rising prevalence of osteoporosis and chronic disease among older adults. However, regional data on surgical timing and its determinants remain limited. Available Saudi studies have primarily focused on epidemiology, fracture patterns, and mortality outcomes, with relatively little emphasis on system-level causes of delay or pathway performance [2,3,17,18]. Moreover, variability in hospital resources, trauma service organization, and access to multidisciplinary care across regions may contribute to inconsistent adherence to international standards, underscoring the need for locally generated evidence.

Understanding the relative contribution of patient-related, provider-related, and system-related factors to surgical delay is essential for designing targeted interventions that are both feasible and effective within the Saudi healthcare context [18-20]. A systems-based analytical approach, rather than a purely clinical perspective, is particularly important in identifying modifiable bottlenecks and informing policy-level decisions, such as the implementation of dedicated hip fracture pathways, optimization of operating room scheduling, and standardization of perioperative processes.

Against this background, the present study was conducted to provide a comprehensive systems analysis of surgical timing in elderly hip fracture patients managed at two major regional hospitals in the Medina Region of Saudi Arabia. By examining surgical timing patterns, identifying predictors of delay, and evaluating the clinical consequences of delayed surgery, this study aims to generate evidence that can inform quality improvement initiatives and support alignment with international best practices in hip fracture care.

## Materials and methods

Methodology

Study Design and Setting

This study was conducted as a retrospective cohort systems analysis evaluating surgical timing and its determinants among elderly patients with hip fractures. The study was carried out at two major public hospitals in the Medina Region of Saudi Arabia, namely King Fahad Hospital and Medina General Hospital. These institutions serve as regional referral centers providing emergency orthopedic trauma care for a large catchment population.

Study Population and Eligibility Criteria

All patients aged 60 years and older who were admitted with a diagnosis of hip fracture and subsequently underwent surgical fixation during the study period were eligible for inclusion. Hip fractures were defined as femoral neck (intracapsular), intertrochanteric, or subtrochanteric fractures confirmed by radiographic imaging. Only patients managed operatively were included to allow assessment of surgical timing and perioperative processes.

Patients were excluded if they had pathological fractures secondary to metastatic disease, sustained polytrauma with competing injuries that independently mandated surgical delay, or were managed non-operatively. Patients with incomplete records regarding admission time or time of surgery were also excluded from analysis. A total population sampling approach was used, and all eligible patients meeting the inclusion criteria during the study period were included, resulting in a final analytic cohort of 400 patients.

Data Sources and Data Collection

Data were extracted retrospectively from hospital electronic medical records and patient charts using a structured data extraction form designed specifically for this study. The extraction process was standardized across both hospitals to ensure consistency and completeness. Collected variables were grouped into patient-level, clinical-level, hospital-level, and system-level domains.

Patient-level variables included age, sex, and comorbidity burden. Comorbidities were summarized using the American Society of Anesthesiologists (ASA) physical status classification, which served as a proxy measure of preoperative physiological risk. Information on the use of anticoagulant or antiplatelet medications at admission was also recorded.

Clinical variables included fracture type, categorized as femoral neck, intertrochanteric, or subtrochanteric, as well as operative details. Time-based variables were recorded in hours or minutes as appropriate and included time of injury (when available), time of hospital admission, time to orthopedic consultation, laboratory turnaround time, and time of surgical incision.

Hospital-level and system-level variables included hospital of admission, weekend or holiday admission status, off-hours admission, inter-facility transfer status, operating room utilization at the time of admission, ward or intensive care unit bed occupancy, anesthetist grade, orthopedic reviewer level, number of preoperative consultations prior to anesthesia clearance, and occurrence of operating room list deferral or cancellation (“bumping”).

Outcome variables included time from admission to surgery, hospital length of stay, postoperative complications, and in-hospital mortality. Postoperative complications were defined as any documented medical or surgical complication occurring during the index hospitalization, including but not limited to pneumonia, deep vein thrombosis, wound infection, or delirium.

Exposure Definition and Group Stratification

The primary exposure variable was surgical timing, defined as the time from hospital admission to surgical fixation. Based on international guidelines and prior literature, patients were stratified into two groups: early surgery, defined as surgery performed within 48 hours of admission, and delayed surgery, defined as surgery performed more than 48 hours after admission. This threshold was selected to reflect widely accepted clinical standards and to allow comparison with international studies.

Statistical Analysis

All statistical analyses were performed using IBM SPSS Statistics for Windows, Version 27 (Released 2019; IBM Corp., Armonk, New York, United States). Continuous variables were assessed for normality using visual inspection and distributional characteristics. Normally distributed continuous variables were summarized as means with standard deviations, whereas non-normally distributed variables were summarized as medians with interquartile ranges. Categorical variables were summarized as frequencies and percentages.

Comparative analyses between early and delayed surgery groups were conducted using appropriate statistical tests. Student’s t-test was used for normally distributed continuous variables, and the Mann-Whitney U test was used for non-normally distributed continuous variables. Chi-square tests were used to compare categorical variables, and Fisher’s exact test was applied when expected cell counts were small. A two-sided p-value of less than 0.05 was considered statistically significant.

To identify independent predictors of surgical delay, multivariable logistic regression analysis was performed with delayed surgery (>48 hours) as the dependent variable. Variables included in the multivariable model were selected based on clinical relevance and statistical significance in univariate analyses. Adjusted odds ratios with 95% confidence intervals were reported. Variables demonstrating perfect prediction, such as operating room list deferral in the early surgery group, were excluded from the regression model because stable parameter estimation was not feasible.

Given the very low number of in-hospital deaths, multivariable regression analysis for mortality was not performed, as such modeling would be statistically unstable. Instead, the association between surgical timing and in-hospital mortality was assessed using Fisher’s exact test, and unadjusted odds ratios were reported for descriptive purposes.

Ethical considerations

Ethical approval for this study was obtained from the Institutional Review Board of Al Rayan National College of Medicine. As this was a retrospective review of existing medical records, the requirement for informed consent was waived. Patient confidentiality was maintained throughout the study by anonymizing data and assigning unique study identification codes. No direct patient contact or intervention occurred, and no conflicts of interest were declared.

## Results

Study population and baseline characteristics

A total of 400 elderly patients with surgically treated hip fractures were included in the final analysis. Most patients underwent surgery beyond the recommended 48-hour window, while a smaller proportion received early surgical intervention. The overall median time from admission to surgery demonstrated a substantial delay in operative management.

Baseline demographic and clinical characteristics according to surgical timing are summarized in Table 1. Overall, the early and delayed surgery groups demonstrated comparable baseline profiles. Patients experiencing delayed surgery tended to be older, although this difference was not statistically significant. Sex distribution, treating hospital, fracture laterality, comorbidity burden, and anticoagulant or antiplatelet therapy use were similar between groups. Differences were observed in ASA classification, while intertrochanteric fractures were more frequently represented among delayed cases. As expected, time from admission to surgery was substantially shorter among patients treated within the early surgery group.

**Table 1:** Baseline characteristics and surgical timing (overall and by timing group) KFH: King Fahad Hospital; MGH: Medina General Hospital; ASA: American Society of Anesthesiologists

Characteristic	Overall (N=400)	Early ≤48h (n=94)	Delayed >48h (n=306)	Statistical test	Test statistic	p-value
Age, years	77.0 ± 8.3	74.7 ± 7.8	77.7 ± 8.3	Mann–Whitney U	U = 11263.0	0.001
Sex				Chi-square	χ²(1) = 0.720	0.396
Male	164 (41.0%)	35 (37.2%)	129 (42.2%)			
Female	236 (59.0%)	59 (62.8%)	177 (57.8%)			
Hospital				Chi-square	χ²(1) = 2.034	0.154
KFH	287 (71.8%)	62 (66.0%)	225 (73.5%)			
MGH	113 (28.2%)	32 (34.0%)	81 (26.5%)			
Fracture type				Chi-square	χ²(2) = 5.078	0.079
Femoral neck	166 (41.5%)	45 (47.9%)	121 (39.5%)			
Intertrochanteric	222 (55.5%)	44 (46.8%)	178 (58.2%)			
Subtrochanteric	12 (3.0%)	5 (5.3%)	7 (2.3%)			
ASA class				Chi-square	χ²(3) = 12.418	0.006
I	31 (7.8%)	15 (16.0%)	16 (5.2%)			
II	160 (40.0%)	36 (38.3%)	124 (40.5%)			
III	199 (49.8%)	42 (44.7%)	157 (51.3%)			
IV	10 (2.5%)	1 (1.1%)	9 (2.9%)			
Any comorbidity				Chi-square	χ²(1) = 1.339	0.247
No	97 (24.2%)	27 (28.7%)	70 (22.9%)			
Yes	303 (75.8%)	67 (71.3%)	236 (77.1%)			
Any anticoagulation/antiplatelet				Chi-square	χ²(1) = 0.185	0.667
No	38 (9.5%)	10 (10.6%)	28 (9.2%)			
Yes	362 (90.5%)	84 (89.4%)	278 (90.8%)			
Time from admission to surgery (hours)	85.7 (54.3–132.5)	35.6 (18.9–45.9)	97.0 (74.5–140.8)	Mann–Whitney U	U = 1965.0	<0.001

System-level and provider-level factors associated with delay

System- and provider-related variables are presented in Table 2. Several healthcare process factors contributed to delays in surgical timing. Weekend or holiday admission was more commonly associated with delayed surgery, suggesting a potential impact of healthcare system workflow on timely operative care. In contrast, off-hours admission, inter-facility transfer, operating room utilization, and ward or ICU occupancy showed no meaningful differences between groups.

**Table 2:** System- and provider-level factors associated with surgical delay (>48 hours)

Factor	Overall (N=400)	Early ≤48h (n=94)	Delayed >48h (n=306)	Statistical test	Test statistic	p-value
Weekend/holiday admission				Chi-square	χ²(1) = 12.631	<0.001
No	292 (73.0%)	82 (87.2%)	210 (68.6%)			
Yes	108 (27.0%)	12 (12.8%)	96 (31.4%)			
Off-hours admission				Chi-square	χ²(1) = 0.579	0.447
No	161 (40.2%)	41 (43.6%)	120 (39.2%)			
Yes	239 (59.8%)	53 (56.4%)	186 (60.8%)			
Transfer from another hospital				Fisher exact	—	0.035
No	365 (91.2%)	91 (96.8%)	274 (89.5%)			
Yes	35 (8.8%)	3 (3.2%)	32 (10.5%)			
OR utilization (%)	75.0 (50.0–100.0)	75.0 (13.2–100.0)	75.0 (56.2–100.0)	Mann–Whitney U	U = 13422.0	0.300
Ward/ICU occupancy (%)	75.0 (50.0–75.0)	75.0 (75.0–75.0)	75.0 (50.0–75.0)	Mann–Whitney U	U = 14256.0	0.885
Lab turnaround time (min)	40.0 (21.5–61.0)	40.0 (20.0–50.0)	43.0 (22.0–65.0)	Mann–Whitney U	U = 12209.0	0.026
Time to orthopedic review (min)	60.0 (20.0–104.0)	60.0 (12.5–97.0)	60.0 (20.0–104.0)	Mann–Whitney U	U = 14765.0	0.694
First reviewer level				Chi-square	χ²(2) = 3.693	0.158
Resident	289 (72.2%)	62 (66.0%)	227 (74.2%)			
Registrar	16 (4.0%)	3 (3.2%)	13 (4.2%)			
Consultant	94 (23.5%)	29 (30.9%)	65 (21.2%)			
Consultant present in theatre				Not estimable	—	—
No	0 (0.0%)	0 (0.0%)	0 (0.0%)			
Yes	400 (100.0%)	94 (100.0%)	306 (100.0%)			
Anesthetist grade				Fisher exact	—	1.000
Consultant	399 (99.8%)	94 (100.0%)	305 (99.7%)			
Specialist	1 (0.2%)	0 (0.0%)	1 (0.3%)			
Number of consults before clearance	3.0 (2.0–3.0)	2.0 (2.0–3.0)	3.0 (2.0–3.0)	Mann–Whitney U	U = 10198.5	<0.001
Bumped from OR list				Chi-square	χ²(1) = 25.615	<0.001
No	331 (82.8%)	94 (100.0%)	237 (77.5%)			
Yes	69 (17.2%)	0 (0.0%)	69 (22.5%)			

Laboratory turnaround time demonstrated a trend toward longer processing times among delayed cases, whereas time to orthopedic assessment was similar between groups. The level of the first orthopedic reviewer and anesthetist grade were not associated with surgical timing. Patients who experienced delayed surgery required more preoperative specialty consultations, and operating room list cancellation or deferral was identified as an important contributor to delay.

Clinical outcomes according to surgical timing

Clinical outcomes are summarized in Table 3. Delayed surgery was associated with increased hospital length of stay compared with early surgical intervention. However, postoperative complication rates and in-hospital mortality were not significantly different between the two groups, suggesting that the main observed impact of delayed surgery was prolonged hospitalization rather than increased short-term adverse events.

**Table 3:** Outcomes by timing group (impact of delay)

Outcome	Overall (N=400)	Early ≤48h (n=94)	Delayed >48h (n=306)	Statistical test	Test statistic	p-value
Length of stay (days)	8.0 (6.0–10.0)	6.0 (4.0–8.0)	8.0 (7.0–10.0)	Mann–Whitney U	U = 8534.0	<0.001
Any postoperative complication				Chi-square	χ²(1) = 3.270	0.071
No	360 (90.0%)	80 (85.1%)	280 (91.5%)			
Yes	40 (10.0%)	14 (14.9%)	26 (8.5%)			
In-hospital mortality				Fisher exact	—	0.236
No	396 (99.0%)	92 (97.9%)	304 (99.3%)			
Yes	4 (1.0%)	2 (2.1%)	2 (0.7%)			

Multivariable predictors of surgical delay

The multivariable regression analysis identifying independent predictors of surgical delay is shown in Table 4. After adjustment for demographic, clinical, and healthcare system variables, increasing age, intertrochanteric fracture pattern, weekend or holiday admission, longer laboratory turnaround time, and a greater number of preoperative consultations remained independently associated with delayed surgery. Differences between hospital sites were also observed after adjustment.

**Table 4:** Multivariable logistic regression: independent predictors of delay (>48 hours) KFH: King Fahad Hospital; MGH: Medina General Hospital; ASA: American Society of Anesthesiologists

Predictor	Adjusted OR	95% CI	p-value
Hospital: MGH (vs KFH)	0.29	0.11–0.75	0.011
Male (vs Female)	1.3	0.64–2.64	0.464
Fracture: Intertrochanteric (vs Femoral neck)	2.07	1.01–4.24	0.048
Fracture: Subtrochanteric (vs Femoral neck)	0.2	0.03–1.54	0.122
Age (per 1 year)	1.04	1.01–1.08	0.015
ASA III–V (vs I–II)	1.1	0.53–2.28	0.792
Any anticoag/antiplatelet (yes vs no)	0.28	0.05–1.62	0.155
Weekend/holiday admission (yes vs no)	2.84	1.11–7.23	0.029
Off-hours admission (yes vs no)	1.13	0.55–2.33	0.744
Inter-facility transfer (yes vs no)	2.67	0.50–14.21	0.250
OR utilization at admission (per 1%)	1	0.98–1.02	0.775
Ward/ICU occupancy at admission (per 1%)	1.01	0.99–1.04	0.337
Lab turnaround (per 1 min)	1.02	1.00–1.03	0.012
Time to orthopedic review (per 1 min)	1	0.99–1.00	0.125
Number of consults (per 1 consult)	1.88	1.33–2.66	<0.001

Other factors, including sex, ASA classification, anticoagulant use, admission timing, transfer status, operating room utilization, and hospital occupancy measures, were not independent predictors of delay. Operating room list cancellation or deferral was excluded from the final model because of statistical limitations related to complete prediction of delayed surgery.

Mortality analysis

Due to the low number of in-hospital deaths, adjusted multivariable mortality analysis was not feasible. The comparison shown in Table 5 demonstrated no significant association between surgical timing and in-hospital mortality.

**Table 5:** Fisher’s exact test: p = 0.527 unadjusted odds ratio (delayed vs early surgery): 0.56

Group	Deaths, n/N (%)
Early ≤48h	2/88 (2.3%)
Delayed >48h	3/312 (1.0%)

## Discussion

In this regional systems analysis of 400 elderly hip fracture patients managed at King Fahad Hospital and Medina General Hospital, most patients underwent surgery beyond the widely accepted early window. The median admission-to-surgery interval was approximately four days, and only a minority received surgery within 48 hours. This finding is clinically important because international guidance consistently treats hip fracture as a time-critical condition, recommending expedited operative management as part of a coordinated pathway. The National Institute for Health and Care Excellence (NICE) hip fracture guideline emphasizes early surgery delivered through a multidisciplinary hip fracture programme to accelerate recovery and reduce complications [15]. Similarly, the Association of Anaesthetists’ guideline and British Orthopaedic Association Standards for Trauma (BOAST) align around facilitating surgery within 36 hours for most patients, with delays reserved for active, reversible medical instability [5,9]. Against this benchmark, the magnitude of delay observed in our cohort indicates substantial opportunities for pathway redesign and prioritization.

Our data suggest that delays were driven predominantly by modifiable organizational and process factors rather than immutable patient characteristics. Although ASA class differed between timing groups on unadjusted comparison, the multivariable model did not identify ASA as an independent predictor of delay. Instead, delay was independently associated with weekend/holiday admission, prolonged laboratory turnaround time, and an increasing number of preoperative consultations. These patterns closely match “avoidable delay” constructs described in international literature, where theatre access, process inefficiencies, and repeated preoperative steps frequently account for the majority of late operations. A recent analysis explicitly categorized most hip fracture surgical delays as avoidable and attributed the largest share to operating theatre availability, highlighting the scope for improvement through systems interventions [19]. In our cohort, the “bumped from the OR list” variable showed complete separation, with no early-surgery patients experiencing bumping and approximately one-quarter of delayed patients being bumped; this provides strong internal evidence that theatre scheduling and prioritization, rather than patient readiness alone, are critical bottlenecks. While we excluded bumping from standard logistic regression because perfect prediction prevents stable maximum-likelihood estimates, the directionality and clinical meaning are unambiguous and support theatre-capacity solutions such as protected trauma lists or ring-fenced hip-fracture slots.

Weekend admission emerged as an independent predictor of delayed surgery, consistent with “weekend effects” reported in multiple health systems where staffing patterns, access to diagnostics, and operating room availability differ on weekends and holidays. In pathway-driven systems, the weekend effect is mitigated by standardized, seven-day services for orthogeriatrics, anesthesia, imaging, and theatre access. National audit and guideline frameworks in the United Kingdom were built partly to reduce such variability by setting time-to-surgery standards and monitoring compliance through structured reporting (e.g., the National Hip Fracture Database model) [4,5]. Our findings suggest that similar measurement and accountability mechanisms, adapted to the Saudi context, could help standardize performance across days of the week and reduce inequities driven by admission timing.

The association between a higher number of consultations and delay likely reflects two interacting phenomena: (i) genuine medical optimization in frail patients and (ii) process fragmentation, where sequential “clearances” occur without parallelization and without a shared risk framework for proceeding to theatre. Internationally, contemporary guidance emphasizes that optimization should occur rapidly and in parallel, and that surgery should proceed unless the patient has an immediately reversible condition that materially changes perioperative risk, because the harms of prolonged immobilization are substantial [9]. The observation that laboratory turnaround time independently predicted delay further reinforces the presence of process latency. In high-performing hip fracture pathways, preoperative blood tests, electrocardiography, imaging, and anesthesia assessment are typically executed early in the admission timeline through protocolized order sets and dedicated hip fracture teams; delays arising from routine investigations are generally viewed as targets for quality improvement rather than accepted “clinical necessity” [7,9].

We also found that the intertrochanteric fracture pattern was independently associated with delay. While fracture type can influence operative planning, implant availability, and surgeon scheduling, most modern pathways do not regard fracture subtype as a justification for prolonging time to theatre, particularly when standardized implants and trauma theatre access are available. One plausible explanation is that intertrochanteric fractures in our cohort may have been associated with higher physiological stress (e.g., pain, blood loss, anemia) or logistical factors (e.g., implant or device availability, competing trauma cases). This association underscores the importance of ensuring implant readiness and streamlined preoperative optimization for extracapsular fractures. The finding is also noteworthy in the Saudi literature, where extracapsular/peritrochanteric fractures have been linked with higher mortality risk, emphasizing the need to avoid compounding baseline risk with system delay [2].

Delayed surgery in our cohort was strongly associated with a longer hospital length of stay, even though postoperative complication rates and in-hospital mortality did not differ significantly between early and delayed groups. The length-of-stay signal is consistent with broader evidence indicating that surgical delay increases resource utilization and delays mobilization and discharge planning. A Canadian study reported that fixation beyond 48 hours was associated with longer length of stay and higher mortality at one year, reinforcing that timeliness has downstream effects beyond the immediate perioperative period [14]. Importantly, our mortality analysis was constrained by a very low number of in-hospital deaths, which limits statistical power and makes multivariable mortality modeling unstable. The absence of a detectable mortality difference in-hospital should therefore not be interpreted as evidence that timing is unimportant; instead, it likely reflects limited event counts and the possibility that any survival effect emerges over longer follow-up windows (e.g., 30-day or one-year mortality), as shown in many large cohort studies. In the international literature, earlier surgery (often within 24-48 hours) has repeatedly been associated with lower mortality and fewer pressure ulcers, including a large meta-analysis encompassing over 190,000 patients [13]. A population-level Italian study similarly found lower one-year mortality among patients undergoing surgery within two days, even after accounting for confounding and survivorship to day two [6]. More recent work argues that a 36-hour target may confer stronger mortality benefits than a 48-hour target, supporting guideline convergence on 36 hours in some jurisdictions [9,12]. Taken together, our length-of-stay findings align with established evidence that timeliness is consequential, and our mortality findings should be interpreted cautiously given follow-up duration and low event rates.

From a Saudi perspective, our findings align with the pattern of health-system gaps increasingly highlighted in regional syntheses. A recent Saudi systematic review identified delayed surgical care and system-level practice variability as recurring challenges and explicitly called for locally adapted standardized care pathways and improved timeliness [3]. Qualitative work from Saudi hospitals has also emphasized implementation barriers to best-practice post-hip-fracture care, such as early mobilization and weight-bearing, which are downstream processes that depend heavily on timely surgery, coordinated multidisciplinary teams, and standardized protocols [21]. Within western Saudi Arabia, a retrospective cohort reported substantial one-year mortality and identified peritrochanteric fractures as a higher-risk subgroup, reinforcing the imperative to optimize the entire pathway for vulnerable patients rather than accept prolonged waits as inevitable [2]. In this context, the prominent role of weekend admission, consult burden, laboratory latency, and OR list disruptions in our cohort provides actionable targets for Saudi institutions seeking to implement evidence-based hip fracture pathways.

The practical implication of our analysis is that improving timeliness in Medina region hospitals will likely require pathway-level interventions rather than isolated clinical reminders. International experience suggests that bundled, multidisciplinary models are effective when they explicitly address the bottlenecks we observed: seven-day access to anesthesia and orthogeriatrics, protocolized investigations and optimization, early parallel processing rather than sequential clearance, and protected theatre capacity for hip fractures. Orthogeriatric unit models and multidisciplinary care pathways have been shown to reduce time to surgery and length of stay in other settings, supporting a strategy of pathway implementation rather than ad hoc case-by-case management [17,22]. While the exact configuration should be adapted to local staffing and hospital resources, the consistent international message is that reliable access to theatre and standardized multidisciplinary workflows are central to achieving early surgery targets.

This study has limitations that should guide interpretation and inform future work. The retrospective design means that unmeasured confounding may persist, particularly around clinical instability variables not fully captured by ASA class or comorbidity indicators. The low in-hospital mortality rate limited our ability to assess mortality effects robustly, and longer follow-up (30-day and one-year mortality) would be more sensitive to timing-related differences. Additionally, the strong association between OR list bumping and delay could not be quantified in standard logistic regression due to complete separation, although the descriptive signal strongly implicates theatre scheduling constraints as a key driver. Future analyses would benefit from prospective pathway metrics (time to imaging, time to anesthesia assessment, time to medical optimization completion, and explicit “avoidable vs unavoidable” delay categorization) and inclusion of post-discharge outcomes.

## Conclusions

In summary, our findings demonstrate a substantial surgical timing gap in elderly hip fracture care within the Medina region, with delays largely explained by system and process factors rather than patient factors alone. The association between delay and prolonged length of stay indicates meaningful operational and likely clinical consequences. Aligning local practice with international hip fracture pathway standards, through protected theatre access, seven-day multidisciplinary services, and streamlined preoperative processes, represents a practical and evidence-supported route to improving timeliness and efficiency in Saudi hip fracture care.
